# The influence of extracellular tissue on neutrophil function and its possible linkage to inflammatory diseases

**DOI:** 10.1002/iid3.472

**Published:** 2021-06-11

**Authors:** Richard F. Kraus, Michael A. Gruber, Martin Kieninger

**Affiliations:** ^1^ Department of Anaesthesiology University Medical Centre Regensburg Regensburg Germany

**Keywords:** extracellular, inflammatory disease, matrix, neutrophil function, tissue

## Abstract

**Background:** Migration, production of reactive oxygen species (ROS), release of myeloperoxidase (MPO), and NETosis are functional immunological reactions of elementary importance for polymorphonuclear neutrophils (PMN).

Unregulated inflammatory response of PMN within tissues plays a key role in the pathophysiology of several diseases. However, little is known about the behavior of PMN after migration through blood vessel walls. Therefore, we investigated the influence of the extracellular matrix (ECM) on PMN function.

**Materials and Methods:** We established an in vitro chemotaxis model of type I and III collagen, fibrin, and herbal agarose tissues using µ‐slide chemotaxis devices and *N*‐formylmethionine‐leucyl‐phenylalanine (fMLP). PMN within the matrices were assessed with a fluorescent time‐lapse microscope for live‐cell imaging.

**Results:** PMN function was obviously influenced by the ECM. Type III collagen had an inhibitory effect on PMN migration regarding track length, direction, and targeting. Type III collagen also had an accelerating effect on neutrophil ROS production. Agarose had an inhibitory effect on MPO release and fibrin a retarding effect on NETosis.

**Conclusion:** Because of the high abundance of type III collagen in lung and skin matrices, the interaction of PMN with the respective matrix could be an important mechanism in the pathophysiology of acute respiratory distress syndrome and pyoderma gangrenosum.

## Graphical abstract

Our study compares the impact of different extracellular matrices on neutrophil function. By examining cells with live‐cell imaging in an in vitro model, we showed that type III collagen inhibits neutrophil migration and boosts neutrophil reactive oxygen species production. Furthermore, we found NETosis to be retarded in fibrin and myeloperoxidase release to be delayed in agarose. Our findings contribute to a better understanding of neutrophil regulatory activity in tissues and may provide a better understanding of neutrophils' important role in the development and maintenance of inflammatory phenomena in tissues

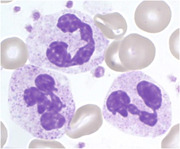

## 1 INTRODUCTION

**Figure 1:**
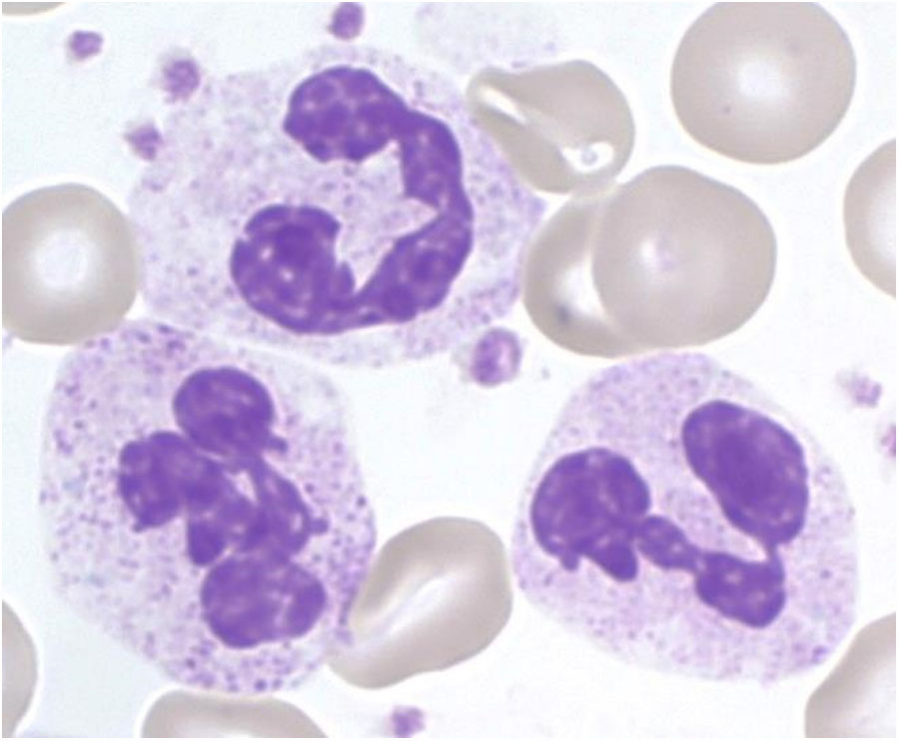
Segmented neutrophil granulocytes in Pappenheim‐stained blood cell smears (graphic provided by the laboratory for Paediatric Oncology and Haematology at the University Medical Centre Regensburg)

Neutrophil granulocytes (polymorphonuclear cells; PMN) constitute about 50%–70% of all circulating leukocytes and are the most mobile and abundant cellular component of the innate immune system of the human body (see Figure 1). PMN are the most important first line of defense within the innate immune response.^1^, ^2^

PMN are rapidly recruited from the bone marrow and transferred to the tissues by targeted chemotaxis. On the way to their target, PMN have to cross the blood vessel walls including the endothelial cell layer and the basement membrane to reach the target tissue after completion of diapedesis.^2^, ^3^, ^4^

Chemotactic migration, production of reactive oxygen species (ROS), release of myeloperoxidase (MPO) and NETosis are among the key functions of the PMN immune defense in tissues. These key functions must be subject to strict regulation because both neutropenia and PMN overreactions constitute alarming and sometimes life‐threatening conditions.^3^

Excessive PMN response is attributed to have a negative effect on the course of certain inflammatory diseases such as acute respiratory distress syndrome (ARDS), cerebral apoplexy, acute coronary syndrome, COVID‐19, or sepsis.^5^, ^6^, ^7^ Furthermore, although several autoimmune diseases are not directly caused by a malfunction of PMN, these cells are known to significantly contribute to disease pathogenesis.^8^ Thus, dysregulated PMN immune response is assumed to have an important impact on autoimmune diseases such as systemic lupus erythematodes (SLE), rheumatoid arthritis (RA) or pyoderma gangrenosum (PG). In most cases, the cause of illness is dysregulation of ROS production, MPO release or NET formation.^9^, ^10^, ^11^

Although PMN are certainly not the only harmful factors in pathophysiological processes, they can assume key roles in the development and maintenance of such processes.^5^

The migration and immune response of granulocytes have been the subject of intensive research for decades. The more advanced the progress in this respect, the clearer it has become that it is difficult to integrate all important in vivo parameters in a single setup.^12^ Because of its more realistic environment, so far a three‐dimensional test setup has been found to be the best option for simulating the conditions in the human body as accurately as possible.^13^ A new analysis technology was described, among others, by Hattenkofer et al.^14^ in 2018 and by Weckmann et al.^15^ in 2017, that allowed the investigation of the migration of PMN at the single cell level in a three‐dimensional space. However, only PMN migration was investigated by means of these analyses. A methodological approach that was first published by Doblinger et al.^16^ in 2019 and proposed as a standard method by Pai et al.^17^ in 2020 enables the simultaneous analysis of both migration and immune effects in one assay. For the first time, the simultaneous consideration of the elementary PMN functions migration, ROS production, MPO release, and NET formation allowed a combined function analysis, even though the phagocytosis aspect was not included. In several studies, the times of maximum ROS production (T_max_ROS) and half‐maximal effect of MPO release and NETosis proved to be suitable parameters for comparing PMN function.^16^, ^17^, ^18^, ^19^

Although several previous studies such as by Oakes et al.^20^ investigated the influence of individual extracellular matrix (ECM) components on PMN migration, none of them simultaneously investigated the influence of the ECM on PMN migration as well as neutrophil immune effects.

To simulate human body environments as accurately as possible, previous studies embedded PMN in a gel matrix similar to that present in human tissue.^21^, ^22^ Depending on the body site to be simulated, the matrix mainly consisted of type IV collagen to imitate the conditions present in endothelial basement membranes, as done by Weckmann et al.^15^ for example. Reid et al.^23^, ^24^ used a matrix consisting of type I collagen to imitate the interstitial ECM because this type of collagen is the most common fibrinous component of the ECM. Fibrin gels, used for example by Moghe et al.,^25^ are well suited for the reconstruction of blood clot models in the inflammatory phase of wound healing or for stroma in solid tumour growth. In addition, chemotaxis studies using agarose as a gel matrix have been reported in the literature.^21^ Such “under‐agarose assays,” as described by Nelson et al.^26^ for instance, use agarose matrices because of their good laboratory properties, even though plant‐based agarose is not present in the human body.

Despite the diverse use of different gel matrices in previous PMN chemotaxis studies, little is known about how the ECM influences PMN. In view of the fact that misdirected PMN activation can depend to a large extent on activities taking place in the interstitium, the present work showed the influence of the PMN environment, that is, the surrounding interstitial matrix, on PMN functions.^27^ The types of different gel matrices used were chosen to reflect the composition, elasticity, and density of the barriers and tissues that migrating PMN meet in the human body.^28^

## 2 MATERIALS AND METHODS

### 2.1 Vote of the Ethics Committee

The conduct of this study was approved by the local Ethics Committee of the Medical Faculty of the University of Regensburg (file number 16‐101‐0322).

### 2.2 Sample collection and isolation of neutrophil granulocytes

Granulocytes were isolated from the blood of healthy donors by means of density gradient centrifugation (LeukoSpin/LymphoSpin, pluriSelect) and resuspended in Roswell Park Memorial Institute (RPMI)‐1640 culture medium (Pan Biotech) and 10% fetal calf serum (Sigma‐Aldrich) at a concentration of 18 × 10^6^ cells/ml, as described by Doblinger et al.^16^

### 2.3 Preparation of the chemotaxis chamber

µSlide chemotaxis chambers® (IBIDI GmbH) were used for the chemotaxis investigations.^14^, ^15^, ^16^, ^29^ At each of the three positions, these chambers have a channel in the middle, which was filled with the respective cell‐gel matrix (see Section 2.4) in a liquid state. At the beginning of our experiments, the total average of migrating neutrophils in each extracellular matrix was generally 110 cells (*T* = 0 min). To the left and right of this channel, there are two reservoirs. After hardening of the matrix, the left reservoir was filled with a 10 nM fMLP solution (Sigma). Analogously, the RPMI‐1640 medium was added to the right reservoir. The two components *ibidi Heating System* and *ibidi Gas incubation System for* CO_2_ (IBIDI) generated a test atmosphere with 37°C, 5% CO_2_, and 50% air humidity for live‐cell imaging (see Section 2.5).

### 2.4 Preparation of the different three‐dimensional extracellular matrices

A type I collagen matrix in a concentration of 1.5 mg/ml was prepared according to the protocol *Application Note 26* (IBIDI).^14^, ^16^, ^30^ For gel preparation, type I collagen (Advanced Biomatrix) in a concentration of 3.0 mg/ml was used and mixed with buffer and cell suspension. A matrix with type III collagen in a concentration of 1.0 mg/ml was prepared by dissolving 10 mg type III collagen (Merck) in 3.33 ml 0.5 M acetic acid (Sigma‐Aldrich) and mixing with buffer and cell suspension. An agarose gel matrix in a concentration of 2.5 mg/ml was prepared according to the method described by Foxman et al.^31^ For this purpose, an agarose solution of 10 mg/ml (Sigma‐Aldrich) was mixed with a buffer solution and heated to 66°C. For the preparation of a fibrin gel in a concentration of 3 mg/ml, the two main components fibrinogen (5 mg/ml; Merck) and thrombin (2 U/ml) (Merck) were pipetted together based on the method described by Moghe et al.^25^ The components of the gel matrices are listed in Table 1.

**Table 1:** Ingredients of the gels used for functional PMN testing

Gel component	Vendor		Type I collagen (1.5 mg/ml)	Agarose (2.5 mg/ml)	Type III collagen (1.0 mg/ml)	Fibrin (3.0 mg/ml)
Cell suspension		(µl)	50	75	25	50
3.0 mg/ml type I collagen	Adv. Biomatrix	(µl)	150	‐	‐	‐
3.0 mg/ml type III collagen	Merck	(µl)	‐	‐	50	‐
10 mg/ml agarose	Sigma‐Aldrich	(µl)	‐	50	‐	‐
MEM	Sigma‐Aldrich	(µl)	20	‐	10	‐
RPMI‐1640	Pan‐Biotech	(µl)	50	35.5	25	138
7.5%‐NaHCO_3_	Sigma‐Aldrich	(µl)	10	‐	7.5	‐
H_2_O distilled		(µl)	20	‐	10	‐
NaOH	Sigma‐Aldrich	(µl)	‐	‐	22.5	‐
HBSS	Sigma‐Aldrich	(µl)	‐	35.5	‐	‐
Fetal calf serum	Sigma‐Aldrich	(µl)	‐	4	‐	‐
Fibrinogen (5 mg/ml)	Merck	(µl)	‐	‐	‐	300
Thrombin (25 U/ml)	Merck	(µl)	‐	‐	‐	12

Abbreviations: HBSS, Hanks' balanced salt solution; MEM, minimum essential medium; PMN, polymorphonuclear neutrophils; RPMI‐1640, Roswell Park Memorial Institute 1640 medium.

### 2.5 Microscopy and live cell imaging

Migration and fluorescence were measured according to Doblinger et al. using a DMi8 inversion microscope (Leica Microsystems), and photographic images were obtained using a DFC9000 GT SCMOS black and white camera (Leica Microsystems). Control of the microscope and the observation process was computer‐aided using the Application Suite X software platform (Leica Microsystems).

The total observation time of each experiment under the microscope was 6 h. In each cycle, four images at ×100 magnification per IBIDI channel were acquired at intervals of 30 s, so that the processes of each IBIDI channel were recorded in 720 individual images. ROS production was assessed by means of 1 μM dihydrorhodamine 123 (DHR; Thermo Fisher Scientific).^16^, ^32^ NETosis was visualized using 5 µM 4′,6‐diamidino‐2‐phenylindole (Sigma‐Aldrich) and MPO release using 0.5 μg/ml anti‐MPO‐APC antibodies (Miltenyi Biotech).^16^, ^33^, ^34^ For fluorescence microscopy, we used excitation filters between 380–410 and 472–498 nm, emission filters between 424–460 and 505–545 nm, and dichroic beam splitters at 418 and 502 nm (Chroma Technology Corp.). Migration was observed in phase contrast (see Table 2).^17^

**Table 2:** Overview of fluorescent‐microscopic detection properties of the different immune effects

Effect	Colorant	Wave length of excitation/emission
NETosis	DAPI	385 nm/461 nm
MPO release	ANTI‐MPO‐APC	635 nm/660 nm
ROS production	rhodamine 123	490 nm/532 nm
Migration	./.	phase contrast

Abbreviations: DAPI, 4′,6‐diamidino‐2‐phenylindole; MPO, myeloperoxidase; NET, neutrophil extracellular trap; ROS, reactive oxygen species.

### 2.6 Evaluation of the microscope images

The image series generated by the microscope were analyzed using the Imaris® 9.0.2 computer software (Bitplane AG). To quantify migration, axes in x and y direction were defined for each channel, as in a Cartesian coordinate system, over the entire length of the considered image area so that defined migration quantities could be determined (see Table 3). The filter track displacement length (TDL) > 10 µm was set.

**Table 3:** Parameters recorded for quantifying migration via live‐cell imaging

TrackDisplacement X (µm)	Covered distance in direction x
TrackDisplacement Y (µm)	Covered distance in direction y
TrackLength (µm)	Effectively covered distance
TrackDisplacement Length (µm)	Euclidean distance between the start and the end point
TrackStraightness $=\frac{\text{TrackDisplacementLength}}{\text{TrackLength}}$	Measure for directionality of movement
TrackDuration (s)	Duration of “tracked” chemotactic migration

The 30‐min observation intervals were normalized to the time of gel–cell contact. Due to the curing time, the nomenclature was chosen in such a way that the first observation period, which started 30 min after cell contact, was designated “0–30,” and the nomenclature was then continued accordingly.

ROS production was assessed by determining the time of maximum ROS production (T_max_ROS) using Excel (Microsoft Corporation) as described before.^16^ To establish the average level of ROS production of single neutrophil cells over 6 h, luminous intensities of ROS‐producing cells were quantified by Imaris® parameter “IntensityMeanROS” and averaged in 30‐min intervals in each IBIDI channel. These average levels of luminous intensities were then summed up over 6 h. MPO release and NETosis were evaluated by determining the time of half‐maximal effect size (ET_50_MPO and ET_50_NETosis) using Phoenix® 8.0.0 (Certara L.P.) as previously described.^16^ The time of first contact between PMN and gel matrix was chosen as a uniform reference point.

### 2.7 Statistical analysis

Statistical analysis was done using SPSS® Statistics 25 (IBM). Normal distribution was verified by the Kolmogorov–Smirnov test. With normal distribution and in case of multiple comparisons and existing homogeneity of variance, the mean values (MV) were checked for significant differences by a single‐factor analysis of variance (ANOVA) with indication of the standard deviation ± *SD*. If there was no homogeneity of variance, Welch's ANOVA test was applied. With existing homogeneity of variance, the subsequent post‐hoc analysis was done according to Bonferroni. In case of existing inhomogeneity of variance, Dunnet′s T3 test was used. The test for homogeneity of variance was conducted using Levene's test. Simple or grouped boxplots were used for the graphical representation of normal distribution. If there was no normal distribution in the data to be compared, the central tendencies of the individual groups were compared with Kruskal–Wallis testing, indicating the median and the interquartile range. The post‐hoc analysis after this test was conducted by Dunn‐Bonferroni testing. An error probability of *p* < .05 was considered statistically significant.

## 3 RESULTS

### 3.1 Type III collagen inhibits neutrophil movement and reinforces neutrophil ROS production

The impact of the ECM on neutrophil migration, ROS production, MPO release and NETosis was assessed with a fMLP chemotaxis gradient in several series of experiments using different matrices consisting of type I and III collagen, fibrin, and agarose according to the model described by Doblinger et al.^16^ For measurement of neutrophil mobility, neutrophil migration length (track length) was determined in intervals of 30 min over 3 h and normalized to the time point of the first gel contact of the neutrophils. Our results showed that in all types of gels, the determined track lengths diminished with increasing duration of the experiment. The total number of single moving neutrophil cells (tracks) decreased with increasing observation time from interval to interval (see Table 4).

**Table 4:** Overview of medians an interquartile ranges of migration lengths (TrackLength) of the different types of gel (left column) in determined 30 min time sections, which could be observed by means of live‐cell imaging

	0–30	31–60	61–90	91–120	121–150	151–180
Type I collagen	250.4 µm (205.5 µm) [*n* = 1280]	201.7 µm (131.1 µm) [*n* = 1115]	147.0 µm (131.8 µm) [*n* = 739]	118.1 µm (106.0 µm) [*n* = 267]	92.8 µm (87.7 µm) [*n* = 112]	83.6 µm (66.6 µm) [*n* = 77]
Agarose	170.1 µm (154.8 µm) [*n* = 889]	150.1 µm (135.5 µm) [*n* = 933]	97.4 µm (71.1 µm) [*n* = 555]	88.9 µm (63.0 µm) [*n* = 513]	81.0 µm (34.5 µm) [*n* = 290]	84.7 µm (33.6 µm) [*n* = 246]
Type III collagen	108.2 µm (82.9 µm) [*n* = 373]	90.1 µm (55.2 µm) [*n* = 257]	61.2 µm (54.9 µm) [*n* = 118]	71.4 µm (94.8 µm) [*n* = 37]	110.6 µm (83.6 µm) [*n* = 18]	66.6 µm (54.6 µm) [*n* = 18]
Fibrin	175.4 µm (199.7 µm) [*n* = 431]	196.7 µm (130.7 µm) [*n* = 427]	104.1 µm (89.5 µm) [*n* = 366]	49.3 µm (50.9 µm) [*n* = 107]	35.0 µm (28.9 µm) [*n* = 20]	28.7 µm (17.4 µm) [*n* = 12]

*Note*: Data are shown as median (IQR) from *n* = 9 experiments per gel type (In each table cell, the uppermost value without brackets indicates the median, the value in round brackets the interquartile range and the value in square brackets contains the number of recorded tracks).

**Figure 2:**
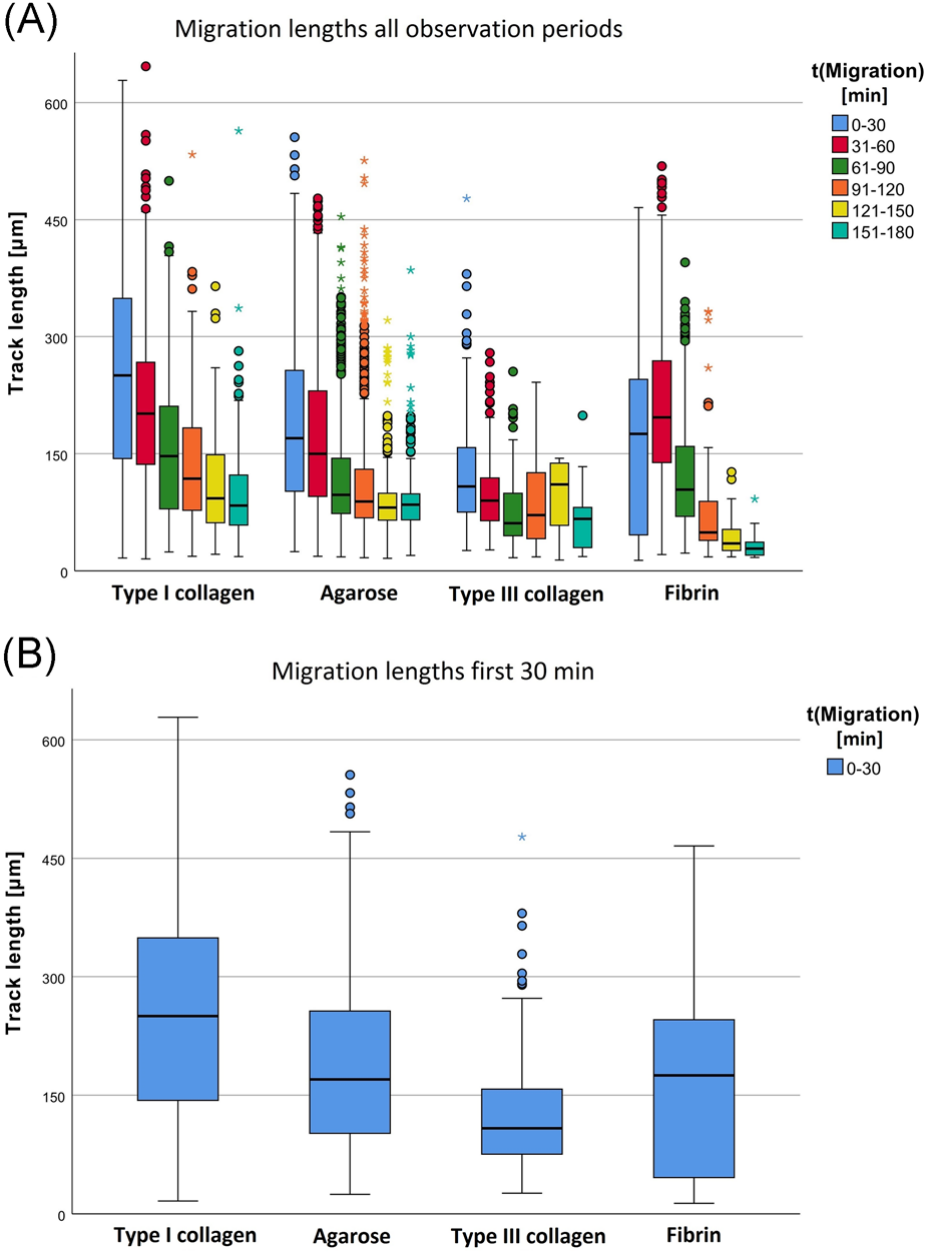
Different neutrophil migration patterns in different extracellular matrices determined by live‐cell imaging. (A) Overview of migration distances of PMN (TrackLength [µm]), split in observation periods of 30 min, and types of gel normed to the first gel contact. In all types of gel, migration decreased towards the end of the observation period. The grouped box plot presents the results of *n* = 9 tests per gel type, using medians with a confidence interval. (B) Different migration lengths (TrackLength [µm]) in the first observation section “0–30”; the shortest migration length was found for type III collagen. Data are shown as median with a confidence interval from nine independent experiments per gel type

In type III collagen, migration was lower than in the other gels (see Table 4 and Figure 2). This observation was most evident in the first period after gel contact because the medians of TL differed between all types of gels (*p* < .001). The results of the other time periods were similar to the results of the first period and showed comparable significant findings. Thus, a significant influence of ECM on neutrophil mobility could be determined.

**Figure 3:**
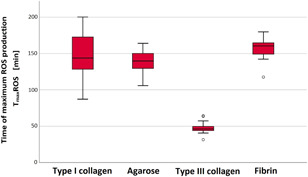
Significantly premature reactive oxygen species (ROS) production in type III collagen. The time of maximum ROS production (T_max_ROS) was determined by live‐cell imaging. Data are shown as median with a confidence interval of T_max_ROS from *n* = 9 experiments per gel type

In addition to migration length, we also investigated the influence of the ECM on neutrophil ROS production. For this purpose, the time of maximum ROS production (T_max_ROS) was determined for the different types of gels. T_max_ROS was significantly premature in type III collagen compared to the other gels (see Table 5 and Figure 3). A significant influence of the ECM on neutrophil ROS production could be determined because of the significant mean differences in T_max_ROS in type III collagen in comparison to the other gel types (*p* < .001).

**Table 5:** Overview of immune effect parameters determined by live‐cell imaging

Type of gel	*T*_max_ROS (min)/(*n*)	ET_50_MPO (min)/(*n*)	ET_50_NETosis (min)/(*n*)	$\frac{T_{\max}\mathrm{ROS}}{{\mathrm{ET}}_{50}\mathrm{NETosis}}\times 100$(%)	$\frac{{\mathrm{ET}}_{50}\mathrm{MPO}}{{\mathrm{ET}}_{50}\mathrm{NETosis}}\times 100$(%)	$\frac{T_{\max}\mathrm{ROS}}{{\mathrm{ET}}_{50}\mathrm{MPO}}\times 100$(%)
Type I collagen	148.1 ± 32.1/(14)	204.2 ± 76.4/(11)	251.6 ± 59.0/(15)	58.9	81.2	72.5
Agarose	138.7 ± 15.1/(16)	267.1 ± 21.4/(12)	246.6 ± 33.3/(17)	56.2	108.3	52.0
Type III collagen	47.9 ± 7.1/(22)	155.1 ± 52.9/(13)	186.2 ± 94.5/(20)	25.7	83.3	30.9
Fibrin	157.0 ± 14.3/(17)	165.4 ± 25.9/(12)	287.3 ± 41.9/(17)	54.6	57.7	95.0

*Note*: Data are shown as mean ± standard deviation (min) from *n* = 9 experiments per gel. The number of experiments (*n*) specifies the number of successful analyzable µ‐slide channels. Quotient formation shown in the right half of the table provides a relative measure for the time gap between immune effects.

Abbreviations: ET_50_MPO, time of half‐maximal release of myeloperoxidase; ET_50_NETosis, time of half‐maximal netosis effect; T_max_ROS, time of maximum ROS production

Mean T_max_ROS of type I collagen significantly differed from that in type III collagen with a mean difference (MD) of 100.2 ± 8.7 min (*p* < .001). Mean T_max_ROS of type III collagen also differed significantly from that of agarose with an MD of 90.8 ± 4.1 min (*p* < .001) and that of fibrin with an MD of 109.1 ± 3.8 min (*p* < .001). In addition, a significant difference in mean T_max_ROS was found for agarose and fibrin with an MD of 18.3 ± 5.1 min (*p* = .007), whereby T_max_ROS was seen at an earlier time in agarose than in the other gels.

**Figure 4:**
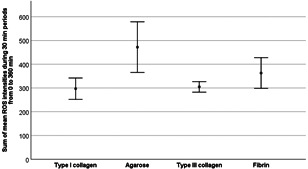
Significantly increased reactive oxygen species (ROS) production in single neutrophil cells in agarose averaged over 6 h. The sum of mean ROS intensities was determined by live‐cell imaging. Data are shown as mean ± 95% confidence interval of the sum of mean ROS intensities from *n* = 9 experiments per gel type

To further investigate the influence of ECM on the amount of ROS production in single cells, we determined the average luminous intensities of ROS production in single neutrophil cells for 6 h (sum of mean ROS intensity, see Figure 4).

The comparison of the sums of mean ROS intensities in the different gels showed that the average luminous intensity of a single cell was significantly higher in agarose than in collagen I (*p* = .025) or collagen III (*p* = .028). The average intensity level of fibrin did not show any significant difference and was between the levels of the different collagens and that of agarose.

### 3.2 Agarose has an inhibitory effect on MPO release

**Figure 5:**
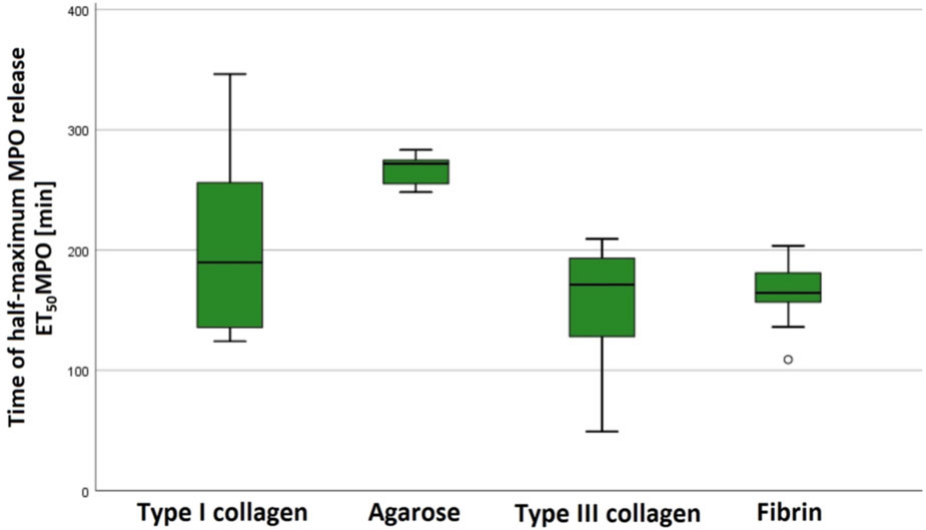
Significantly decelerated semimaximum effect of myeloperoxidase (MPO) release in agarose. Time of half‐maximal netosis effect (ET_50_MPO) was determined by live‐cell imaging. Significant differences were observed between agarose and type III collagen as well as between agarose and fibrin. Data are shown as median with a confidence interval of ET_50_MPO from *n* = 9 experiments per gel type

Next, we investigated the impact of the ECM on neutrophil MPO release. Determination of the time point of the half maximum effect of MPO release (ET_50_MPO) showed a significant influence of the ECM on neutrophils (see Table 5 and Figure 5). Mean ET_50_MPO occurred significantly later in agarose than in type III collagen with an MD of 112.1 ± 15.9 min (*p* < .001). Mean ET_50_MPO occurred significantly later in agarose than in fibrin with an MD of 101.7 ± 9.7 min (*p* < .001).

### 3.3 Fibrin has an inhibitory effect on neutrophil NETosis

**Figure 6:**
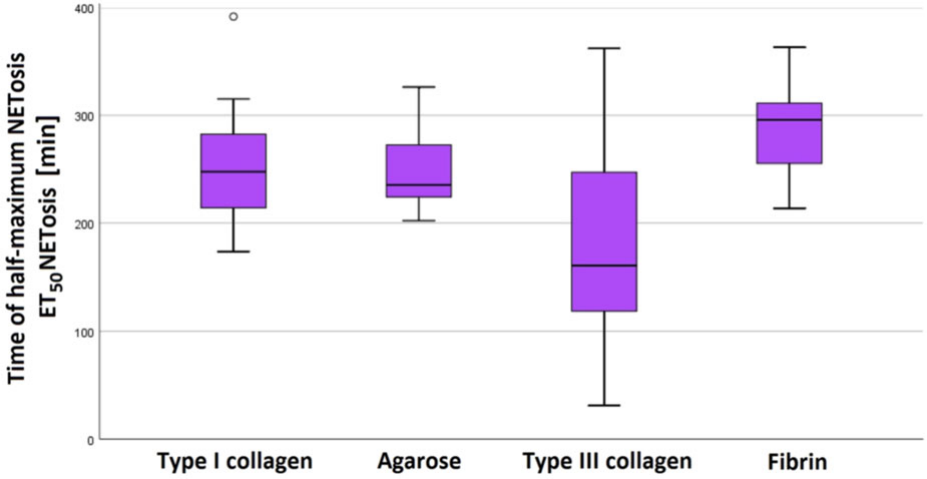
Significant retardation of the half‐maximum effect of NETosis in fibrin. Time of half‐maximal netosis effect (ET_50_NETosis) was determined using live‐cell imaging. Significant differences were found between agarose and fibrin as well as between type III collagen and fibrin. Data are shown as median with a confidence interval of ET_50_NETosis from *n* = 9 experiments per gel type

Finally, we analyzed the impact of the ECM on neutrophil NETosis. The time of the half‐maximum effect (ET_50_NETosis) was determined for the respective gels. All migrating cells initiated NETosis up to the end of the observation period. In fibrin, NETosis was retarded in comparison to agarose (with an MD of 40.8 ± 13.0 min, *p* = .022). In fibrin, NETosis was also retarded in comparison to type III collagen with an MD of 101.2 ± 23.4 min (*p* = .001; see Figure 6).

### 3.4 Presence of a categorical schedule of neutrophil defensive mechanisms but no fixed timeline

Our experiments presented the sequence of immune effects ROS production, MPO release and NETosis in matrices of type I and III collagen as well as fibrin. In agarose, we observed a deviating sequence with NETosis before MPO release. Through formation of quotients of the single times of the immune effects (see Table 5, right half), we obtained a relative measure of the immune effects to each other. The resulting possible assessment of the time relation indicated rather heterogeneous temporal relations in the different types of gels (see Table 5).

### 3.5 ECM influences the direction and targeting of neutrophil movement

**Figure 7:**
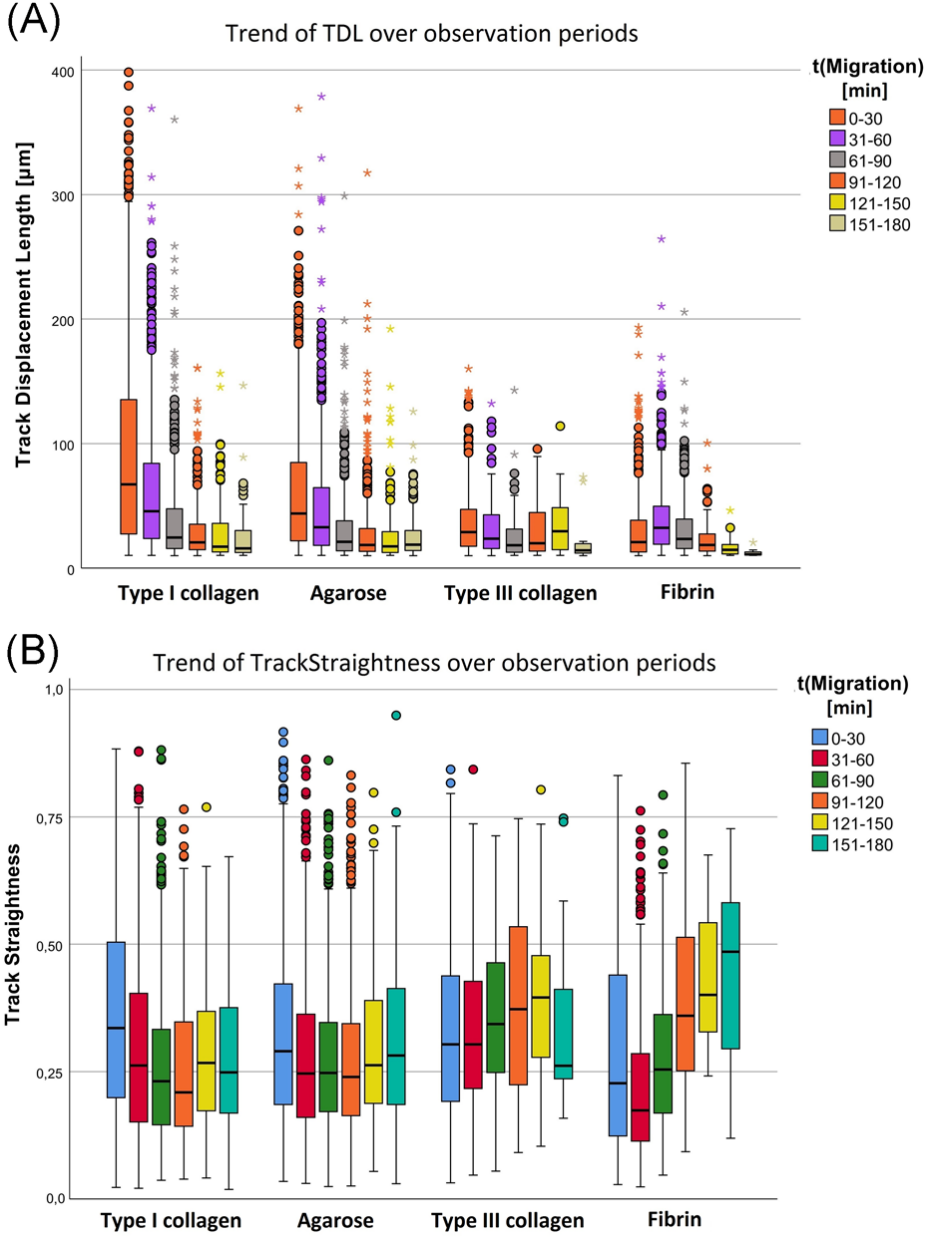
Impact of the ECM on the direction and targeting of PMN migration. (A) Euclidean distance (track displacement length [TDL]) was determined by means of live‐cell imaging. Significant differences in the first observation section indicated a directional influence of the ECM. Data are shown as median with a confidence interval from *n* = 9 experiments per gel type. (B) Significant dependence of PMN migration targeting from ECM. TrackStraightness was determined with live‐cell imaging. In the first observation section “0–30,” PMN moved most target‐oriented in type I collagen. In the last observation section “151–180,” PMN moved most target‐oriented in fibrin. Data are shown as median with a confidence interval from *n* = 9 experiments per gel type

Finally, we conducted a more detailed analysis of the neutrophil course of movement by evaluating the Euclidean distance (TDL) and track straightness, which was defined as the quotient of the Euclidean distance and the migration length (see Table 6 and Figure 7). In the first observation section, significant differences between the means of TDL of every type of gel indicated a directional influence of the ECM (*p* < .001; except for type III collagen and fibrin: *p* = .003). Significant differences in track straightness of all time sections indicated dependence of movement targeting (*p* < .001). In the first observation section (0–30), PMN moved most target‐oriented in type I collagen and least target‐oriented in fibrin (*p* < .001). This trend reversed towards the last time section (151–180) (*p* < .001).

**Table 6:** Medians and interquartile ranges of the parameter TDL in the period “0–30” determined by means of live‐cell imaging

Type of gel	Type I collagen	Agarose	Type III collagen	Fibrin
Time period “0–30”	67.3 µm (IQR = 108.1 µm)	43.7 µm (IQR = 62.9 µm)	29.0 µm (IQR = 29.6 µm)	20.8 µm (IQR = 25.4 µm)

*Note*: Data are shown as medians ± IQR from *n* = 9 experiments per gel type.

Abbreviations: IQR, interquartile range; TDL, track displacement length.

### 3.6 A chemotaxis gradient was established in every type of gel

The values found for TrackDisplacementX and TrackDisplacementY signalized main neutrophil movement with targeting in the positive X direction, which corresponded to the same direction as the fMLP gradient. The medians of TDX—with exception of the TDX value in fibrin in the last time section (151–180), had a positive value (Table S7). The medians of the parameter TDY were around point zero with values from −2.7 to +3.3 µm within the first four time sections. In the last two time sections, type III collagen showed values from –9.3 to +4.3 µm in contrast to –2.3 to +1.0 µm for the other gels (see Table S8).

## 4 DISCUSSION

### 4.1 PMN activation by type III collagen

The results of this study show the impact of the ECM on neutrophil function. Because of in comparison consistently lower migration lengths and a very early standstill of the PMN (rapid decline of the tracked cells from *n* = 118 (61–90) to *n* = 37 (91–120) and *n* = 18 (121–150), type III collagen seemed to have the strongest inhibitory effect on PMN migration throughout all observation phases. With a in comparison significantly earlier mean T_max_ROS value of 47.9 ± 7.1 min, the impact of the ECM on the ROS production of PMN was best visible in type III collagen.

Calculation of the average luminous intensity of ROS by means of the sum of average “IntensityMeanROS” over 6 h enabled the measurement of the total ROS production in a single cell. Although T_max_ROS occurred significantly earlier in collagen III (47.9 ± 7.1 min) than in the other gels, the average ROS production in single neutrophil cells over 6 h was not increased; in contrast, average ROS production in single cells in collagen III was decreased compared to that of agarose (see Figure 4). Hence, although collagen III had shown a previous maximum ROS production of all neutrophils in the channel, total ROS production was not increased in single cells.

Overall, it can be concluded that PMN are put into a different activation state in gel matrices consisting of type III collagen because they increase ROS production and inhibit migration. The results of this study confirm the postulate published by Nathan et al.^35^ as early as 1989 according to which the cytokine‐induced respiratory burst of PMN and thus the ROS production of PMN depends on interactions with the ECM. Although molecular mechanisms are not part of this study, a molecular classification of the observations may nevertheless be discussed.

Sorokin et al.^36^ reported that the ECM and its biochemical composition may provide specific signals to immune cells, thereby determining the ability of cells to promote inflammatory responses. Therefore, it would be conceivable that type III collagen sends signals to PMN, prompting the observed neutrophil behavior listed in Section 3.1. This theory can be supported through an in vitro study published by Nissen et al.,^37^ who stated that the presence of bioactive collagen fragments affects migration and ROS production of PMN.

In this context, Clark and Brugge^38^ described that integrins are able to convert extracellular signals into intracellular processes. Besides the involvement of integrins in cell attachment to ECM (for more details see Section 4.2), integrin‐receptors also transmit signals from the surroundings, and this transmission initiates and sustains vital cellular responses.^27^ Such integrin outside‐in signaling can be triggered by the activation of neutrophil β2‐integrins on ligand‐coated surfaces in the presence of a proinflammatory stimulus (such as fMLP), subsequently leading to respiratory burst and degranulation responses.^35^, ^39^, ^41^

As reported by Leitinger and Hohenester,^42^ different integrin collagen receptors (such as α_1_β_1,_ α_2_β_1_ and α_10_β_1_) can bind to different types of collagen. This way, different signaling pathways may be activated and explain different neutrophil behavior. The expression level of β1 integrins seems to be relatively insensitive to chemo‐attractant stimulation alone and appears to require additional signaling associated with substrate adhesion.^27^

Because the ligation of one type of integrin by matrix proteins modulates the activity of another type, it is also conceivable that the same integrins (as the integrin α_2_β_1_ in case of type I and III collagen) can bind to the ECM of the different gels but subsequently activate different signaling pathways.^42^, ^43^

Furthermore, ECM may activate G‐protein‐coupled receptors (GPCRs) or affect the interaction of fMLP with GPCRs. A common feature of GPCRs is that chemo‐attractants (such as fMLP) strongly activate the chemotactic migration of neutrophils. Nevertheless, chemoattractants (especially formyl‐peptides) trigger neutrophil responses other than chemotaxis, including ROS production and exocytosis of intracellular granules.^39^

Because of different integrin or GPCR influences (or both), collagen III may activate the PKC‐signaling pathway, resulting in the assembly of the nicotinamide adenine dinucleotide phosphate (NADPH) oxidase complex or mitochondrial activation. NADPH and mitochondrial activation ultimately lead to the production of ROS, which may explain premature T_max_ROS in collagen III.^9^, ^32^, ^39^

The PKC pathway (and thus neutrophil immune response) could, therefore, be activated by both GPCRs and integrins. In line with this theory, Rossaint et al.^44^ observed that neutrophils only induce NET formation if simultaneously activated by both integrin‐mediated outside‐in and GPCR signaling.

Besides the PKC‐signaling pathway, the Src‐signaling pathway may be important for the influence of ECM on PMN. Neutrophils were shown to express integrins with an α4‐subunit (such as α4β1, VLA‐4) related to the collagen receptor integrin family. α4‐integrins are able to recognize ECM proteins, whereby a direct cross‐link of α4 integrins triggers the release of superoxide. α4 integrins signal through Src‐family kinases to transduce intracellular signals, mediating a specific release of neutrophil granules and a long‐standing respiratory burst.^39^, ^45^, ^46^

Because PMN, which lack Src‐ and Syk‐family kinases (both considered essential components of integrin‐signaling in neutrophils) predominantly showed normal migration, it should be noted that integrins may use different signaling transduction pathways to trigger adherent activation and migration of neutrophils.^38^, ^39^

In summary, the different neutrophil behavior observed in different matrices is probably due to the complex interaction of the components described above. Even though the role of integrins and GPCRs in neutrophil adhesion and signalling has been clarified to some extent, this complex interaction should be further elucidated in more detail.^47^

Despite the limitations of the model used in the present work that is considered to reflect the (patho‐) physiological reality, the increased ROS production and the inhibited migration in type III collagen may be linked to PMN‐associated diseases such as acute respiratory distress syndrome (ARDS) and pyoderma gangrenosum (PG). It is striking that PMN are significantly involved in the pathology of these two diseases and that both diseases have their focus in type III collagen‐rich tissues (see below).^48^

All forms of PG show subepidermal and dermal infiltrates of morphologically mature PMN; however, such PMN have abnormal chemotaxis and bactericidal properties and are marked by overexpression and dysregulation of integrins.^11^, ^49^, ^50^ The sites of subepidermal and dermal infiltration show abundant type III collagen in the ECM. In the dermo‐epidermal junction zone, the epithelium of the epidermis is connected to the dermis via a chain of adhesion mechanisms. Type III collagen forms the extensive network of the lamina fibroreticularis of the basement membrane, which is located on the basolateral side of the epidermal epithelium and ultimately mediates the anchoring of the epithelium to the subepithelial tissue of the papillary dermis. In contrast, the spatial network of the papillary dermis consists in large part of type III collagen fibrils.^51^

In ARDS, PMN migrate into the lung parenchyma as part of an inflammatory reaction. During the migration process, PMN release ROS and other substances. The release of ROS may contribute to the damage to alveolar structures, which results in higher paracellular permeability that may ultimately lead to acute lung failure.^5^ The fibrils of the alveolar walls and septa mostly consist of type III collagen.^52^, ^53^ Furthermore, as in the skin, the lamina fibroreticularis of the basement membrane is connected to type III collagen fibrils of the interstitium via anchor fibrils.^54^ Pugin et al.^55^ described that intense inflammation and increased synthesis of type III collagen in the alveoli already occur in the early phase of ARDS.

Gade et al.^56^ described a close connection between neutrophil inflammatory diseases of the skin (PG) and the lung (ARDS), even though pulmonary manifestation of PG is a rather rare condition. In 2016, Watanabe et al.^57^ reported that aggressive disease activity in PG may even trigger ARDS.

Although no valid data are available so far, our results may lead to the assumption that PMN in type III collagen may interact with pathophysiological processes of skin (PG) or lung (ARDS). However, the present work cannot provide any proof of this assumption so that further clarification is necessary.

### 4.2 ECM influences the direction and target orientation of migration

The ECM influenced not only the length but also the direction and target orientation of migration. It was striking that TDL values in the first observation phase (0–30) were significantly lower in fibrin than in the other gels. Similar TDL values were found in fibrin and type I collagen (see Table 4) so that PMN migration in fibrin was less directional than in the other gels because of lower TS values (see Figure 7).

Lower TDL and TS values at the beginning of the observation may be due to the fact that the development of the chemotaxis gradient was more difficult and slower in fibrin, a supposition supported by the increasing TS value towards the end of the experiment (see Figure 7). Another explanation may be that, because of the influence of the fibrin ECM, PMN can follow chemotactic gradients to a lesser extent than in ECMs consisting of type I or III collagen or agarose. The latter assumption would confirm the observation described by Burns et al.^54^ that the adhesion properties of different ECM elements (in this case of fibrin) can not only influence speed and migration length but also the direction of leukocyte movement.

Although no molecular mechanisms were considered in the present work, its results can nevertheless be placed in the context of the current scientific literature.

The significant differences in the migration parameters determined by this study seem to contradict the postulate in a Nature review article published by Nourshargh et al.^4^ According to this postulate, leukocytes can distinguish between an integrin‐dependent and an integrin‐independent mode, and their ameboid mode of locomotion is mostly independent from the composition of the ECM. However, as further formulated by Nourshargh et al., it is not yet clear to what extent leukocytes actually use extracellular guidance structures for locomotion. Particularly during interstitial locomotion, leukocytes remain firmly integrated in the tissue through cell‐matrix contacts. Thus, the ECM seems to modulate rather than strictly determine migration. The significantly different migration lengths found in all gels in this study confirm the influence of the ECM on PMN migration.^4^

As Kuntz et al.^58^, ^59^ found out through various additions to collagen matrices, the impact on PMN migration can be based on a spatial steric barrier function of the ECM. On the contrary, it is also conceivable that potentially integrin‐mediated cellular interactions with the different ECMs generate intracellular biochemical signals, which influence cell motility inside the gels.^38^, ^58^

The inhibitory influence of type III collagen on migration length confirms the observation described by Lindbom and Well.^27^ that the chemotaxis of PMN is also influenced by the relative frequency of matrix proteins inside the tissue. Taking this idea further, this finding could mean that abundant type III collagen in the ECM strongly increases the binding strength between integrins and their ligands, thus counteracting motility.^27^

The different migration lengths could be explained not only by the influence on the binding strength of the same integrin subtypes but also by the fact that cell–ECM interaction in the different matrices is mediated by different integrin subtypes or different ECM receptors. Leitinger and Hohenester^42^ described different collagen receptors on immune cells, each of which binds specifically to individual collagen types, which may result in different control of the cell behavior of immune cells. Furthermore, various subtypes of β1 integrins on the surface of PMN have already been described, which bind to some ECM components such as fibronectin but not to other ECM components such as type I or IV collagen. According to Sixt et al.,^60^ such specific integrin‐mediated interactions signal specific information to the cells that can exert control over PMN movement in inflammation foci.

Overall, the results of this study confirm a conclusion formulated by Loike et al. as early as 1995: PMN chemotaxis is regulated by the composition of the ECM.^43^

### 4.3 Sequence of defensive mechanisms

In our experiments, the sequence of the immune effects in type I and III collagen and fibrin gels was as follows: ROS production, MPO release and finally NETosis. This sequence, which is consistent with earlier observations by Doblinger et al., is recognized in the current scientific literature and reflects the fact that ROS und MPO are released through degranulation first, whereas NETosis occurs last.^9^, ^16^, ^61^ The deviating sequence in agarose observed in our experiments can be explained by inaccuracy of measurement methods.

With regard to the quotient $T_{\max}\mathrm{ROS}/{\mathrm{ET}}_{50}\mathrm{NETosis}$, similar relation values were only found for type I and III collagen and fibrin gels. The quotient ${\mathrm{ET}}_{50}\mathrm{MPO}/{\mathrm{ET}}_{50}\mathrm{NETosis}$ also had similar values in type I and III collagen gels. Consequently, the time intervals between the effects varied according to the gel matrix. This finding suggests that there is no fixed, temporally defined scheme according to which these effects occur successively.

Although ROS production seems to be indispensable for NETosis, the two processes do not seem to be directly linked in a consecutive manner. Despite more intensive ROS production of single neutrophil cells, the herb agarose even delayed ET_50_MPO, and ET_50_NETosis was also not determined prematurely (see Section 3.1). In spite of mean ROS intensities similar to those in type I collagen and fibrin, type III collagen seemed to ensure reduction in neutrophil migration lengths. Type III collagen also seemed to effectuate, that most neutrophils produced ROS at an early time point, and both ET_50_NETosis and ET_50_MPO occured at an early stage.

### 4.4 MPO release

The choice of extracellular gel matrix seems to influence the MPO release of PMN. With a mean value of 267.1 ± 21.4 min, MPO release in agarose was delayed compared to that in the other gels. However, these results were only statistically significant when MPO release in agarose was compared to that in type III collagen and fibrin but not in type I collagen.

Similar to excessive ROS production, uncontrolled MPO release by PMN involves the risk of tissue damage, which is associated with a negative course of various inflammatory diseases such as rheumatoid arthritis or acute and chronic pneumonia.^62^ The knowledge of the molecular mechanisms behind inhibited MPO release in agarose would be helpful to develop a drug‐based control of PMN overreaction in acute and chronic inflammatory diseases.

### 4.5 NETosis

We observed a significant impact of ECM gels on PMN NETosis. Gel matrices consisting of fibrin seemed to have an inhibitory effect. With a mean value of 287.3 ± 41.9 min, ET_50_NETosis occurred last in fibrin, and the results were statistically significant in comparison to those of agarose and type III collagen.

Recent studies on sepsis have shown that excessive formation of NETs correlates with the development of organ damage and that NETs are associated with increased disseminated intravascular coagulation (DIC).^63^, ^64^ NETs are also assumed to contribute to an increased tendency to thrombosis in patients with SLE or COVID‐19 as well as to premature filter blockage during the long‐term use of extracorporeal membrane oxygenation (ECMO) in critical care patients.^7^, ^65^, ^66^, ^67^, ^68^

In both infectious and noninfectious diseases, NETs are also formed within the vascular system. A study by Fuchs et al.^65^ showed that NETs can have a procoagulating effect, resulting in the formation of a scaffold. Furthermore, Semeraro et al.^69^ reported, that extracellular histones (important components of NETs) promote thrombin generation. On the one hand, NETs also have a stimulating effect regarding the adhesion, activation and aggregation of thrombocytes. On the other hand, NETs promote thrombin‐dependent fibrin generation by binding fibrinogen. NETs and fibrin have been observed to colocalise in vitro. On the basis of these findings, Fuchs et al.^65^ hypothesized that NETs closely interact with fibrin strands in the thrombus, thus influencing thrombus organization and stability.

In summary, NETs and NETosis promote the formation and stabilization of fibrin thrombi; fibrin, however, inhibits NETosis and thus the formation of NETs. If this idea is consistently pursued, a negative feedback mechanism may be assumed. This assumption raises the question whether there is a fibrin‐triggered NETosis delay mechanism and thus ultimately a fibrin‐controlled delay mechanism of NETosis‐mediated thrombosis. Although earlier approaches (for instance by Bredthauer et al.^18^) failed to prove the influence of drug‐based anticoagulants (heparins and argatroban) on the NETosis of PMN, PMN‐fibrin interaction may be a starting point for inhibiting the coagulation‐promoting effect of PMN.^18^

### 4.6 Characteristics and limitations of the in vitro test model

The development of individual matrices was based on the maxim to create physiologically relevant extracellular gel matrices. Despite the careful selection of the applied methods, the study has some limitations.

To be able to also observe T_max_ROS in type III collagen under the microscope, the polymerization time was reduced to 15 min, and the observation periods had to be adjusted. Without this adjustment, evaluations would have been incorrect because different periods would have been compared with each other with respect to the reference point. Several studies, for instance, by Chenoweth et al.^70^ and Trevani et al.,^71^ have shown that the pH value of the extracellular environment may influence PMN functions. Therefore, attention was paid to adjusting the pH value to the neutral point, using primarily the CO_2_/HCO_3_‐buffer system because this system plays the most important physiological role in extracellular buffering in the human body.

According to a study by Trevani et al.,^71^ the extracellular pH value alone neither increases ROS production nor MPO release. However, PMN release more hydrogen peroxide in acidic environments in the additional presence of fMLP. It could be argued that the pH value was not properly adjusted to a neutral value during the production of the type III collagen matrix and that premature T_max_ROS (see Table 5) was solely due to this fact. However, this assumption is disproved by the fact that, according to Trevani et al., an acidic pH value without the presence of fMLP does not increase ROS production. Furthermore, Trevani et al. showed that PMN have a significantly delayed apoptosis and an extended lifetime in acidic environments.^71^ However, our experiments showed that NETosis in type III collagen occurs earlier (with a mean ET_50_NETosis value of 186.2 ± 94.5 min) than in other gels (cf. Section 4.5). From a critical point of view, premature T_max_ROS in type III collagen could be explained by the fact that cells in type III collagen came into contact with the chemokine fMLP 15 min earlier than the cells in the other gels. However, in consideration of the mean values of T_max_ROS and ET_50_NETosis, the significant mean value differences diverge by more than 15 min (cf. Table 5). In addition, this consideration should take into account that, according to Kim snd Wu,^72^ it takes about 80 min to reach a steady‐state of a fMLP gradient, which in turn relativises an fMLP contact of 15 min earlier. Furthermore, we considered the time of first contact between PMN and the gel matrix as the appropriate reference time point because it proved to be the least susceptible time to interference because of the lowest standard deviation in comparison and because the influence of the ECM on PMN was effective from this point in time onwards.

Finally, we would like to note, that due to the influence of the ECM, comparisons of experimental results regarding the function of PMN (particularly migration, ROS production, MPO release, and NETosis) should be considered critically when PMN have been embedded in different gel matrices.

## 5 CONCLUDING REMARKS

PMN play a key role in the development and maintenance of pathophysiological processes in inflammatory diseases.^5^ Given that misdirected PMN activations may depend to a high degree on activities taking place in the interstitium, the results of this study showed an influence of the ECM on the neutrophil functions migration, ROS production, MPO release, and NETosis.

Type III collagen inhibited neutrophil migration and increased neutrophil ROS production. Taking into consideration that PMN‐associated diseases such as PG and ARDS have lesion foci in ECMs consisting of type III collagen‐rich tissue, the question arises whether increased neutrophil ROS production and inhibited migration in ECMs consisting of type III collagen are associated with excessive PMN responses in inflammatory diseases. The knowledge of the molecular mechanisms behind inhibited MPO release in agarose would also be helpful to develop a drug‐based control of PMN overreaction in acute and chronic inflammatory diseases.

The inhibitory effect of fibrin on NETosis could be based on a negative feedback mechanism as a regulatory measure in case of increased NET‐mediated thrombus formation. If the existence of such a feedback could be confirmed, the molecular mechanisms could be used to develop substances that delay or prevent PMN‐mediated thrombotic complications such as ECMO filter blockage, thromboses in SLE, or DIC in sepsis.

The results showed that ECM gels influenced not only the length of migration but also the direction of movement. Integrin‐mediated interaction between ECM and PMN would be in accordance with previous studies^27^, ^42^, ^60^ but could not be proven within the scope of this study.

The investigation and manipulation of integrin‐mediated cell–matrix interactions is not only a worthwhile subject in basic research but already an important pillar in the current treatment of inflammatory diseases.^73^ Therefore, for the purpose of therapy‐oriented research, it would be desirable to further investigate the interactions of PMN with their environment to elucidate potential integrin involvement. A better understanding of the molecular modes of action of the ECM influence on PMN could pave the way for a broader use of already established anti‐inflammatory therapies and contribute to the discovery and further development of alternative ways and means of manipulating inflammatory responses.^4^, ^36^

## CONFLICT OF INTERESTS

The authors declare that there are no conflict of interests.

## Supporting information

Supporting information.

## Data Availability

Data available on request from the authors due to privacy/ethical restrictions.

## References

[1] Quinn MT , DeLeo FR . Preface. In: Quinn MT , DeLeo FR , eds. Neutrophil Methods and Protocols. 2nd ed. Totowa, NJ: Humana Press; 2014:vii‐viii.

[2] Kolaczkowska E , Kubes P. Neutrophil recruitment and function in health and inflammation. Nat Rev Immunol. 2013;13(3):159‐175. 23435331 10.1038/nri3399

[3] Sadik CD , Kim ND , Luster AD . Neutrophils cascading their way to inflammation. Trends Immunol. 2011;32(10):452‐460. 21839682 10.1016/j.it.2011.06.008 PMC3470857

[4] Nourshargh S , Hordijk PL , Sixt M. Breaching multiple barriers: leukocyte motility through venular walls and the interstitium. Nat Rev Mol Cell Biol. 2010;11(5):366‐378. 20414258 10.1038/nrm2889

[5] Segel GB , Halterman MW , Lichtman MA . The paradox of the neutrophilˈs role in tissue injury. J Leukoc Biol. 2011;89(3):359‐372. 21097697 10.1189/jlb.0910538 PMC6608002

[6] Shen X‐F , Cao K , Jiang J‐P , Guan W‐X , Du J‐F . Neutrophil dysregulation during sepsis: an overview and update. J Cell Mol Med. 2017;21(9):1687‐1697. 28244690 10.1111/jcmm.13112 PMC5571534

[7] Leppkes M , Knopf J , Naschberger E , et al. Vascular occlusion by neutrophil extracellular traps in COVID‐19. EBioMedicine. 2020;58(102925):1‐9. 10.1016/j.ebiom.2020.102925 PMC7397705 32745993

[8] Benard S , *Chemisches Signal und Biologische Antwort: Modulation der Generierung reaktiver Sauerstoffverbindungen aus neutrophilen Granulozyten*. Akademische Verlagsanstalt Engelsdorf, Leipzig, 2000.

[9] Gupta S , Kaplan MJ . The role of neutrophils and NETosis in autoimmune and renal diseases. Nat Rev Nephrol. 2016;12(7):402‐413. 27241241 10.1038/nrneph.2016.71 PMC5510606

[10] Bardoel Bart W , Kenny Elaine F , Sollberger G , Zychlinsky A. The balancing act of neutrophils. Cell Host Microbe. 2014;15(5):526‐536. 24832448 10.1016/j.chom.2014.04.011

[11] Alavi A , French LE , Davis MD , Brassard A , Kirsner RS . Pyoderma Gangrenosum: an update on pathophysiology, diagnosis and treatment. Am J Clin Dermatol. 2017;18(3):355‐372. 28224502 10.1007/s40257-017-0251-7

[12] Toetsch S , Olwell P , Prina‐Mello A , Volkov Y. The evolution of chemotaxis assays from static models to physiologically relevant platforms. Integr Biol (Camb). 2009;1(2):170‐181. 20023801 10.1039/b814567a

[13] Parkhurst MR , Saltzman WM . Quantification of human neutrophil motility in three‐dimensional collagen gels. Effect of collagen concentration. Biophys J. 1992;61(2):306‐315. 1547321 10.1016/S0006-3495(92)81838-6 PMC1260248

[14] Hattenkofer M , Gruber M , Metz S , Pfaehler S‐M , Lehle K , Trabold B. Time course of chemotaxis and chemokinesis of neutrophils following stimulation with IL‐8 or FMLP. Eur J Inflamm. 2018;16:1‐8.

[15] Weckmann M , Becker T , Nissen G , Pech M , Kopp MV . SiMA: A simplified migration assay for analyzing neutrophil migration. Cytometry A. 2017;91(7):675‐685. 28544679 10.1002/cyto.a.23114

[16] Doblinger N , Bredthauer A , Mohrez M , et al. Impact of hydroxyethyl starch and modified fluid gelatin on granulocyte phenotype and function. Transfusion. 2019;59:2121‐2130. 30934131 10.1111/trf.15279

[17] Pai D , Gruber M , Pfaehler S‐M , Bredthauer A , Lehle K , Trabold B. Polymorphonuclear cell chemotaxis and suicidal NETosis: simultaneous observation using fMLP, PMA, H7, and live cell imaging. J Immunol Res. 2020;2020:1‐10. 10.1155/2020/1415947 PMC7448108 32879894

[18] Bredthauer A , Kopfmueller M , Gruber M , et al. Therapeutic anticoagulation with argatroban and heparins reduces granulocyte migration: possible impact on ECLS‐therapy? Cardiovasc Ther. 2020;26:1‐10. 10.1155/2020/9783630 PMC7196999 32405324

[19] Kolle G , Metterlein T , Gruber M , et al. Potential impact of local anesthetics inducing granulocyte arrest and altering immune functions on perioperative outcome. J Inflamm Res. 2021;14:1‐12. 33442284 10.2147/JIR.S275525 PMC7797324

[20] Oakes PW , Patel DC , Morin NA , et al. Neutrophil morphology and migration are affected by substrate elasticity. Blood. 2009;114(7):1387‐1395. 19491394 10.1182/blood-2008-11-191445 PMC2727411

[21] Keenan TM , Folch A. Biomolecular gradients in cell culture systems. Lab Chip. 2007;8(1):34‐57. 18094760 10.1039/b711887b PMC3848882

[22] Islam LN , McKay IC , Wilkinson PC . The use of collagen or fibrin gels for the assay of human neutrophil chemotaxis. J Immunol Methods. 1985;85(1):137‐151. 4078307 10.1016/0022-1759(85)90282-0

[23] Reid G , Lackie J , Gorham S. The behaviour of BHK cells and neutrophil leukocytes on collagen gels of defined mechanical strength. Cell Biol Int Rep. 1990;14(11):1033‐1045. 2126224 10.1016/0309-1651(90)90115-f

[24] Sixt M , Lämmermann T. In vitro analysis of chemotactic leukocyte migration in 3D environments. Methods Mol Biol (Clifton, NJ). 2011;769:149‐165. 10.1007/978-1-61779-207-6_11 21748675

[25] Moghe PV , Nelson RD , Tranquillo RT . Cytokine‐stimulated chemotaxis of human neutrophils in a 3‐D conjoined fibrin gel assay. J Immunol Methods. 1995;180(2):193‐211. 7714334 10.1016/0022-1759(94)00314-m

[26] Nelson RD , Quie PG , Simmons RL . Chemotaxis under agarose: a new and simple method for measuring chemotaxis and spontaneous migration of human polymorphonuclear leukocytes and monocytes. J Immunol. 1975;115(6):1650‐1656. 1102606

[27] Lindbom L , Werr J. Integrin‐dependent neutrophil migration in extravascular tissue. Sem Immunol. 2002;14(2):115‐121. 10.1006/smim.2001.0348 11978083

[28] Jennings RT , Knaus UG . Neutrophil migration through extracellular matrix. Methods Mol Biol (Clifton, NJ). 2014;1124:209‐218. 10.1007/978-1-62703-845-4_13 24504954

[29] Zengel P , Nguyen‐Hoang A , Schildhammer C , Zantl R , Kahl V , Horn E. mu‐Slide Chemotaxis: a new chamber for long‐term chemotaxis studies. BMC Cell Biol. 2011;12:21. 21592329 10.1186/1471-2121-12-21 PMC3118187

[30] IBIDI GmbH . Application note no. 26: fabrication of collagen I gels. Version 2.4. https://ibidi.com/img/cms/support/AN/AN26_CollagenI_protocols.pdf. Accessed May 1 2020.

[31] Foxman EF , Campbell JJ , Butcher EC . Multistep Navigation and the Combinatorial Control of Leukocyte Chemotaxis. J Cell Biol. 1997;139(5):1349‐1360. 10.1083/jcb.139.5.1349 9382879 PMC2140208

[32] Chen Y , Junger WG . Measurement of oxidative burst in neutrophils. Methods Mol Biol (Clifton, NJ). 2012;844:115‐124. 10.1007/978-1-61779-527-5_8 PMC4214271 22262438

[33] Omelon S , Georgiou J , Habraken W. A cautionary (spectral) tail: red‐shifted fluorescence by DAPI–DAPI interactions. Biochem Soc Trans. 2016;44(1):46‐49. 26862187 10.1042/BST20150231

[34] Buhr N , de von Köckritz‐Blickwede M. How neutrophil extracellular traps become visible. J Immunol Res. 2016;2016:4604713. 27294157 10.1155/2016/4604713 PMC4884809

[35] Nathan C , Srimal S , Farber C , et al. Cytokine‐induced respiratory burst of human neutrophils: dependence on extracellular matrix proteins and CD11/CD18 integrins. J Cell Biol. 1989;109(3):1341‐1349. 2475511 10.1083/jcb.109.3.1341 PMC2115779

[36] Sorokin L. The impact of the extracellular matrix on inflammation. Nat Rev Immunol. 2010;10(10):712‐723. 20865019 10.1038/nri2852

[37] Nissen G , Hollaender H , Tang F , et al. Tumstatin fragment selectively inhibits neutrophil infiltration in experimental asthma exacerbation. Clin Exp Allergy. 2018;48(11):1483‐1493. 30028047 10.1111/cea.13236

[38] Clark EA , Brugge JS . Integrins and signal transduction pathways: the road taken. Science. 1995;268(5208):233‐239. 7716514 10.1126/science.7716514

[39] Futosi K , Fodor S , Mócsai A. Neutrophil cell surface receptors and their intracellular signal transduction pathways. Int Immunopharmacol. 2013;17(3):638‐650. 23994464 10.1016/j.intimp.2013.06.034 PMC3827506

[40] Mócsai A , Zhou M , Meng F , Tybulewicz VL , Lowell CA . Syk is required for integrin signaling in neutrophils. Immunity. 2002;16(4):547‐558. 11970878 10.1016/s1074-7613(02)00303-5

[41] Nathan CF . Neutrophil activation on biological surfaces massive secretion of hydrogen peroxide in response to products of macrophages and lymphocytes. J Clin Investig. 1987;80(6):1550‐1560. 2445780 10.1172/JCI113241 PMC442423

[42] Leitinger B , Hohenester E. Mammalian collagen receptors. Matrix Biol. 2007;26(3):146‐155. 17141492 10.1016/j.matbio.2006.10.007

[43] Loike JD , el Khoury J , Cao L , et al. Fibrin regulates neutrophil migration in response to interleukin 8, leukotriene B4, tumor necrosis factor, and formyl‐methionyl‐leucyl‐phenylalanine. J Exp Med. 1995;181(5):1763‐1772. 7722453 10.1084/jem.181.5.1763 PMC2191980

[44] Rossaint J , Herter JM , van Aken H , et al. Synchronized integrin engagement and chemokine activation is crucial in neutrophil extracellular trap‐mediated sterile inflammation. Blood. 2014;123(16):2573‐2584. 24335230 10.1182/blood-2013-07-516484

[45] Pereira S , Zhou M , Mócsai A , Lowell C. Resting murine neutrophils express functional alpha 4 integrins that signal through Src family kinases. J Immunol (Baltimore, MD 1950). 2001;166(6):4115‐4123. 10.4049/jimmunol.166.6.4115 11238661

[46] Hynes RO . Integrins: bidirectional, allosteric signaling machines. Cell. 2002;110(6):673‐687. 12297042 10.1016/s0092-8674(02)00971-6

[47] Erpenbeck L , Gruhn AL , Kudryasheva G , et al. Effect of adhesion and substrate elasticity on neutrophil extracellular trap formation. Front Immunol. 2019;10:2320. 31632402 10.3389/fimmu.2019.02320 PMC6781793

[48] Gameiro A , Pereira N , Cardoso JC , Gonçalo M. Pyoderma gangrenosum: challenges and solutions. Clin Cosmet Investig Dermatol. 2015;8:285‐293. 10.2147/CCID.S61202 PMC4454198 26060412

[49] Ahronowitz I , Harp J , Shinkai K. Etiology and management of pyoderma gangrenosum. Am J Clin Dermatol. 2012;13(3):191‐211. 22356259 10.2165/11595240-000000000-00000

[50] Adachi Y , Kindzelskii L , Cookingham G , et al. Aberrant neutrophil trafficking and metabolic oscillations in severe pyoderma gangrenosum. J Invest Dermatol. 1998;111(2):259‐268. 9699727 10.1046/j.1523-1747.1998.00311.x

[51] Lüllmann‐Rauch R , Paulsen F. Haut und Hautanhangsgebilde. In: Lüllmann‐Rauch R , Paulsen F , eds. Taschenlehrbuch Histologie. 4th ed. Stuttgart, Germany: Thieme; 2012:550‐576.

[52] Raghu G , Striker LJ , Hudson LD , Striker GE . Extracellular matrix in normal and fibrotic human lungs. Am Rev Respir Dis. 1985;131(2):281‐289. 3882034 10.1164/arrd.1985.131.2.281

[53] Shiomi T , Okada Y , Foronjy R , et al. Emphysematous changes are caused by degradation of type III collagen in transgenic mice expressing MMP‐1. Exp Lung Res. 2003;29(1):1‐15. 10.1080/01902140303761 12652812

[54] Burns AR , Smith CW , Walker DC . Unique structural features that influence neutrophil emigration into the lung. Physiol Rev. 2003;83(2):309‐336. 12663861 10.1152/physrev.00023.2002

[55] Pugin J , Verghese G , Widmer MC , Matthay MA . The alveolar space is the site of intense inflammatory and profibrotic reactions in the early phase of acute respiratory distress syndrome. Crit Care Med. 1999;27(2):304‐312. 10075054 10.1097/00003246-199902000-00036

[56] Gade M , Studstrup F , Andersen AK , Hilberg O , Fogh C , Bendstrup E. Pulmonary manifestations of pyoderma gangrenosum: 2 cases and a review of the literature. Respir Med. 2015;109(4):443‐450. 25622759 10.1016/j.rmed.2014.12.016

[57] Watanabe M , Natsuga K , Ota M , Ito K. Pyoderma gangrenosum associated with acute respiratory distress syndrome. Am J Med. 2016;129(2):17‐18. 10.1016/j.amjmed.2015.08.035 26475257

[58] Kuntz RM , Saltzman WM . Neutrophil motility in extracellular matrix gels: mesh size and adhesion affect speed of migration. Biophys J. 1997;72(3):1472‐1480. 9138592 10.1016/S0006-3495(97)78793-9 PMC1184529

[59] van Goethem E , Poincloux R , Gauffre F , Maridonneau‐Parini I , Le Cabec V. Matrix architecture dictates three‐dimensional migration modes of human macrophages: differential involvement of proteases and podosome‐like structures. J Immunol (Baltimore, MD 1950). 2010;184(2):1049‐1061. 10.4049/jimmunol.0902223 20018633

[60] Sixt M , Hallmann R , Wendler O , et al. Cell Adhesion and Migration Properties of β2‐Integrin Negative Polymorphonuclear Granulocytes on Defined Extracellular Matrix Molecules. J Biol Chem. 2001;276(22):18878‐18887. 11278780 10.1074/jbc.M010898200

[61] Brinkmann V , Zychlinsky A. Neutrophil extracellular traps: Is immunity the second function of chromatin? J Cell Biol. 2012;198(5):773‐783. 22945932 10.1083/jcb.201203170 PMC3432757

[62] Aratani Y. Myeloperoxidase: its role for host defense, inflammation, and neutrophil function. Arch Biochem Biophys. 2018;640:47‐52. 29336940 10.1016/j.abb.2018.01.004

[63] Clark SR , Ma AC , Tavener SA , et al. Platelet TLR4 activates neutrophil extracellular traps to ensnare bacteria in septic blood. Nature Med. 2007;13(4):463‐469. 17384648 10.1038/nm1565

[64] Delabranche X , Stiel L , Severac F , et al. Evidence of netosis in septic shock‐induced disseminated intravascular coagulation. Shock (Augusta, GA). 2017;47(3):313‐317. 10.1097/SHK.0000000000000719 27488091

[65] Fuchs TA , Brill A , Duerschmied D , et al. Extracellular DNA traps promote thrombosis. PNAS. 2010;107(36):15880‐15885. 20798043 10.1073/pnas.1005743107 PMC2936604

[66] Birkenmaier C , Dornia C , Lehle K , et al. Analysis of thrombotic deposits in extracorporeal membrane oxygenators by high‐resolution microcomputed tomography. ASAIO J. 2020:1‐7. 10.1097/MAT.0000000000001089 32740353

[67] Steiger T , Foltan M , Philipp A , et al. Accumulations of von Willebrand factor within ECMO oxygenators: potential indicator of coagulation abnormalities in critically ill patients? Artif Organs. 2019;43(11):1065‐1076. 31192471 10.1111/aor.13513 PMC6899554

[68] Wilm J , Philipp A , Müller T. Leukocyte adhesion as an indicator of oxygenator thrombosis during extracorporeal membrane oxygenation therapy? ASAIO J. 2018;64(1):24‐30. 28475562 10.1097/MAT.0000000000000586

[69] Semeraro F , Ammollo CT , Morrissey JH , et al. Extracellular histones promote thrombin generation through platelet‐dependent mechanisms: involvement of platelet TLR2 and TLR4. Blood. 2011;118(7):1952‐1961. 21673343 10.1182/blood-2011-03-343061 PMC3158722

[70] Chenoweth DE , Rowe JG , Hugli TE . A modified method for chemotaxis under agarose. J Immunol Methods. 1979;25(4):337‐353. 372445 10.1016/0022-1759(79)90026-7

[71] Trevani AS , Andonegui G , Giordano M , et al. Extracellular acidification induces human neutrophil activation. J Immunol. 1999;162(8):4849‐4857. 10202029

[72] Kim BJ , Wu M. Microfluidics for mammalian cell chemotaxis. Ann Biomed Eng. 2012;40(6):1316‐1327. 22189490 10.1007/s10439-011-0489-9 PMC3424276

[73] Rosario M , Dirks NL , Milch C , et al. A review of the clinical pharmacokinetics, pharmacodynamics, and immunogenicity of vedolizumab. Clin Pharmacokinet. 2017;56(11):1287‐1301. 28523450 10.1007/s40262-017-0546-0 PMC5648740

