# Supplementary material for: The influence of extracellular tissue on neutrophil function and its possible linkage to inflammatory diseases
Source: Immun Inflamm Dis. 2021 Jun 11;9(4):1237–51. doi: 10.1002/iid3.472 (PMC8589351; doi:10.1002/iid3.472)
Supplement: Supplementary file 1 — Supporting information. [file IID3-9-1237-s001.docx]

Table 7: The movement on the x-plane (TDX) was almost exclusively positive (in the same direction of the fMLP gradient). Values of TDX were determined in different types of extracellular gels (left column). Data are shown as median (IQR) from n=9 experiments per gel type.

|  | **0-30** | **31-60** | **61-90** | **91-120** | **121-150** | **151-180** |
| --- | --- | --- | --- | --- | --- | --- |
| **Type I collagen** | 18.8µm (87.7µm) | 15.8µm  (56.0µm) | 8.1µm  (34.5µm) | 3.3µm  (26.7µm) | 4.7µm  (26.8µm) | 0.4µm  (20.9µm) |
| **Agarose** | 13.2µm  (61.3µm) | 10.7µm  (45.5µm) | 10.2µm  (29.1µm) | 8.6µm  (27.9µm) | 10.2µm  (25.6µm) | 11.6 µm  (27.1µm) |
| **Type III collagen** | 4.1µm  (38.7µm) | 3.7µm  (35.1µm) | 5.2µm  (19.2µm) | 8.2µm  (34.1µm) | 13.4µm  (41.0µm) | 1.9µm  (19.0µm) |
| **Fibrin** | 3.5µm  (34.8µm) | 8.9µm  (40.4µm) | 2.2µm  (33.2µm) | 2.8µm  (26.3µm) | 10.2µm  (21.9µm) | -2.3µm  (18.5µm) |

Table 8: Movement on the y-plane (TDY) fluctuated around point zero. TDY was determined by means of live-cell imaging in different types of extracellular gels (left column). Data are shown as median (IQR) from n=9 experiments per gel type.

|  | **0-30** | **31-60** | **61-90** | **91-120** | **121-150** | **151-180** |
| --- | --- | --- | --- | --- | --- | --- |
| **Type I collagen** | 1.7µm  (60.0µm) | -1.3µm  (45.2µm) | 1.0µm  (29.5µm) | -2.5µm  (26.6µm) | 1.0µm  (22.9µm) | -0.4µm  (25.9µm) |
| **Agarose** | -0.1µm  (36.7µm) | 0.0µm  32.6µm | -0.7µm  (23.5µm) | -1.1µm  (21.7µm) | -1.4µm  (17.6µm) | -0.3µm  (21.2µm) |
| **Type III collagen** | -2.7µm  (30.1µm) | -2.3µm  (25.8µm) | -0.3µm  (25.8µm) | -1.1µm  (21.4µm) | 4.3µm  (15.9µm) | -9.3µm  (13.9µm) |
| **Fibrin** | 1.0µm  (20.6µm) | 3.3µm  (35.0µm) | -0.6µm  (26.5µm) | 0.0µm  (18.4µm) | -2.3µm  (12.4µm) | -2.1µm  (13.0µm) |
